# Revealing the spatiotemporal requirements for accurate subject identification with resting-state functional connectivity: a simultaneous fNIRS-fMRI study

**DOI:** 10.1117/1.NPh.10.1.013510

**Published:** 2023-02-03

**Authors:** Sergio L. Novi, Alex C. Carvalho, R. M. Forti, Fernado Cendes, Clarissa L. Yasuda, Rickson C. Mesquita

**Affiliations:** a University of Campinas, “Gleb Wataghin” Institute of Physics, Campinas, Brazil; b Western University, Department of Physiology and Pharmacology, London, Ontario, Canada; c University of Campinas, Laboratory of Neuroimaging, Campinas, Brazil; d The Children’s Hospital of Philadelphia, Division of Neurology, Philadelphia, Pennsylvania, United States; e Brazilian Institute of Neuroscience and Neurotechnology, Campinas, Brazil; f University of Campinas, School of Medical Sciences, Department of Neurology, Campinas, Brazil

**Keywords:** functional near-infrared spectroscopy, brain fingerprinting, functional magnetic resonance imaging, subject identification, resting-state functional connectivity

## Abstract

**Significance:**

Brain fingerprinting refers to identifying participants based on their functional patterns. Despite its success with functional magnetic resonance imaging (fMRI), brain fingerprinting with functional near-infrared spectroscopy (fNIRS) still lacks adequate validation.

**Aim:**

We investigated how fNIRS-specific acquisition features (limited spatial information and nonneural contributions) influence resting-state functional connectivity (rsFC) patterns at the intra-subject level and, therefore, brain fingerprinting.

**Approach:**

We performed multiple simultaneous fNIRS and fMRI measurements in 29 healthy participants at rest. Data were preprocessed following the best practices, including the removal of motion artifacts and global physiology. The rsFC maps were extracted with the Pearson correlation coefficient. Brain fingerprinting was tested with pairwise metrics and a simple linear classifier.

**Results:**

Our results show that average classification accuracy with fNIRS ranges from 75% to 98%, depending on the number of runs and brain regions used for classification. Under the right conditions, brain fingerprinting with fNIRS is close to the 99.9% accuracy found with fMRI. Overall, the classification accuracy is more impacted by the number of runs and the spatial coverage than the choice of the classification algorithm.

**Conclusions:**

This work provides evidence that brain fingerprinting with fNIRS is robust and reliable for extracting unique individual features at the intra-subject level once relevant spatiotemporal constraints are correctly employed.

## 1 Introduction

Functional neuroimaging has empowered the understanding of brain function continuously and noninvasively under several conditions since the early 1990s. In particular, blood-oxygen-level-dependent (BOLD) functional magnetic resonance imaging (fMRI) and functional near-infrared spectroscopy (fNIRS) share similar hemodynamic origins and reveal brain function by relying on the neurovascular coupling to infer neural activity and extract functional connectivity (FC) maps.^1^^–^^5^ However, the low signal-to-noise ratio (SNR) intrinsic to both modalities ultimately leads to a lack of intra-subject reproducibility, which is enlarged by several confounding factors, including motion artifacts and physiology from noncortical tissues.^6^^–^^11^ To overcome this limitation, researchers have often opted to perform group analysis by gathering data from different subjects based on their shared features. Although this approach increases the overall SNR and eases group comparisons, collapsing data from participants ignores unique individual features that may hold great potential for subject discrimination—a relevant feature that is valuable for several purposes, including clinical applications in which individualized treatments could benefit patient recovery and outcome.^12^^–^^14^

The individual analysis ultimately depends on data reproducibility, which can be quantitatively assessed with test-retest protocols (i.e., experimental designs in which data are acquired from the same participant several times within days or a few weeks). To this end, several test-retest protocols have been previously applied to characterize the variability of fMRI and fNIRS data.^10^^,^^12^^,^^15^^–^^22^ Because healthy brain function is not expected to change significantly at the macroscale within a few days, repeated runs/sessions can help to develop optimal approaches for data acquisition (such as fMRI sequences and fNIRS probe positioning) and for analysis pipelines to yield more reproducible results at the intra-subject level.^9^^,^^10^^,^^22^^–^^26^ Thanks to these efforts, recent methodologies have increased the intra-subject reproducibility to a level that it is possible to accurately identify individuals based on their FC maps, i.e., brain fingerprinting.

Since the pioneering work by Finn et al., novel approaches have improved brain fingerprinting with FC patterns measured with BOLD-fMRI.^12^ For example, Amico and Goñi explored principal component analysis (PCA) for assessing and optimizing individual identification and showed that unique features at the intra-subject level could be better reconstructed in the connectivity domain.^14^ More recently, Venkatesh et al. demonstrated that the geodesic distance between a pair of correlation matrices yields higher individual classification rates than simply employing the Pearson correlation coefficient to compute the similarity across FC maps from different sessions.^27^ The geodesic distance outperforms linear metrics because it does not neglect that correlation matrices lie in a nonlinear space.^27^^,^^28^

Brain fingerprinting with fNIRS consolidates the feasibility of extracting robust and reliable individual neural features from fNIRS-based hemodynamics, paving the way for continuous bedside clinical works focused on single-patient changes in the short term.^29^^,^^30^ A better understanding of what drives subject identification could allow us to differentiate between participant- and session-dependent brain connectivity patterns, which is critical for isolating longitudinal brain changes from spurious fluctuations of the measured signal. Despite its potential, only a few works have attempted to perform brain fingerprinting with fNIRS to date,^31^^,^^32^ which is probably related to the fact that fMRI methods are not directly translated to fNIRS due to the intrinsic noise properties of the latter. Specifically, fNIRS’ high temporal resolution allows for the removal of systemic physiological noise, and it samples the brain faster than the hemodynamic changes and leads to high temporal autocorrelation in the hemoglobin time series.^33^^,^^34^ The autocorrelation violates the statistical assumptions for the Pearson correlation and inflates the correlation coefficients, resulting in spurious FC maps.^35^ In addition, fNIRS signals are contaminated by extracerebral hemodynamics and motion artifacts,^20^^,^^36^^–^^45^ and both confounding factors can profoundly affect the comparison between hemoglobin time series and decrease sensitivity to actual cortical connectivity patterns.^20^^,^^22^^,^^35^

Recent advances in fNIRS processing and analysis have introduced and validated robust methodologies for each of these challenges, opening new doors to properly translating brain fingerprinting protocols to fNIRS.^9^^,^^45^^,^^46^ However, several experimental and methodological concerns—such as the number and location of the optodes, the amount of data, and the classification method—remain unresolved and could be addressed by a robust methodology for identifying subjects.

In the present work, we aimed to analyze the constraints and limitations associated with the problem of brain fingerprinting. To this end, we acquired resting-state fMRI and fNIRS data simultaneously over several runs. For the fNIRS data, we applied a previously developed and validated preprocessing pipeline, which included motion artifact correction and global systemic physiology removal.^45^ We then quantified how subject identification accuracy was affected by the number of runs, the quantity of information provided by the different neuroimaging techniques, and the methodology used to compare the FC maps. Based on the hypothesis that data-driven techniques would benefit more from using different runs from the same individual than similarity metrics, we compared the performance of a linear classifier with standard matrix distance approaches. For fNIRS in particular, we also explored how the combination of different contrasts (i.e., types of hemoglobin) contributes to the problem.

## 2 Methods

### 2.1 Subjects and Experimental Protocol

A total of 29 healthy subjects (26 males, 18 to 34 years old) contributed to this study. All measurements were performed inside the MRI unit. We instructed all participants to close their eyes, stay relaxed, not move, and not focus their thoughts on any specific task. In each participant, we collected six runs of MRI and fNIRS simultaneously for six minutes each. Because MRI requires one structural image (high-resolution, T1-weighted) run, each participant contributed five and six functional measurements of BOLD-fMRI and fNIRS, respectively. The local ethical committee at the University of Campinas (where the experiments were performed) approved the experimental protocol. Participants provided written informed consent before data acquisition.

### 2.2 fMRI Signal Acquisition and Preprocessing

The MRI data were collected on a Philips Achieva 3 T scan with a 32-channel head coil. We acquired one T1-weighted structural image [Repetition Time (TR) = 7 ms, Time to Echo (TE) = 3.2 s] and five functional resting-state scans (180 volumes, TR = 2 s, TI = 900 ms). The BOLD data were preprocessed with the SPM12 and the UF2C toolbox.^47^ We employed a preprocessing pipeline that included normalization, motion artifact correction (framewise displacement (FD) and DVARS), band-pass filtering between 0.009 and 0.08 Hz, stopband attenuation of 50 dB, regressions of white matter, cerebral spinal fluid, and global signal regression.^7^^,^^23^^,^^26^^,^^48^^–^^50^ The FD and DVARS thresholds were 0.5 mm and 5%, respectively.^23^ All runs with less than 4 min without motion artifacts were excluded;^23^^,^^26^^,^^51^ this procedure removed a total of 22 runs across all 29 participants. After the removal of runs, eight participants had less than three good BOLD-fMRI runs and were excluded from further analysis. For fMRI comparison with fNIRS, we parceled the brain cortex into 94 regions of interest (ROIs) using the anatomical automatic labeling approach.^52^ The time series of each ROI was estimated as the average of all voxels within the ROI.

### 2.3 fNIRS Signal Acquisition and Preprocessing

**Fig. 1:**
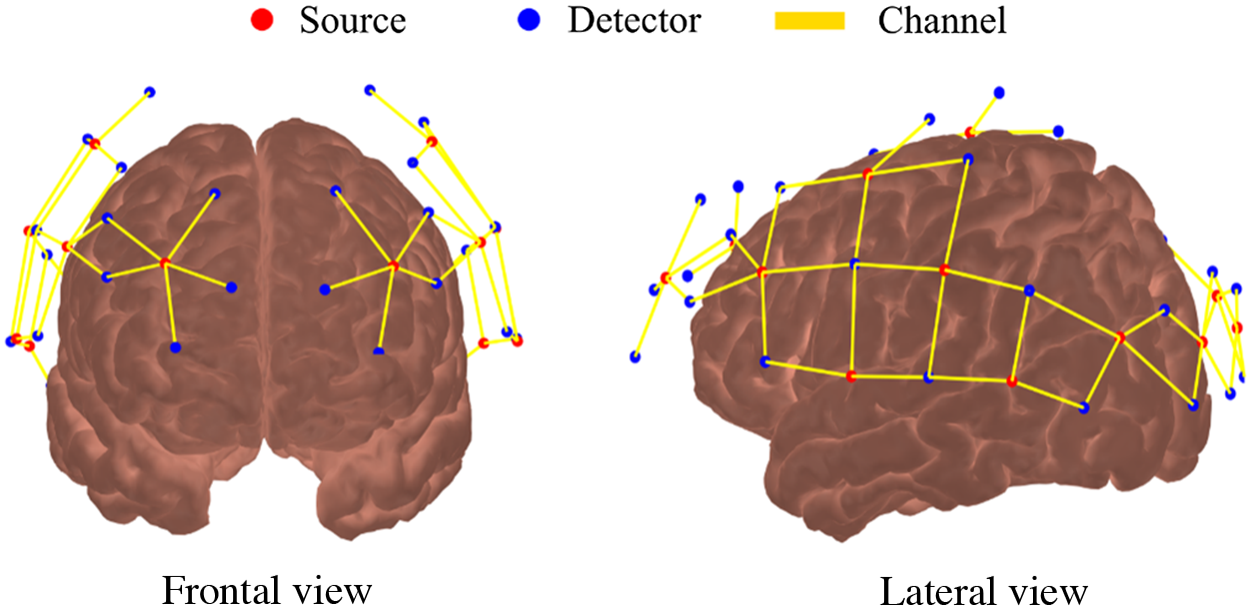
Optical probe used for fNIRS. The probe contains 16 sources (red dots) and 32 detectors (blue dots) distributed symmetrically between the 2 hemispheres, covering most of the head and including frontal, temporal, parietal, and occipital lobes. The solid yellow lines represent the 64 source-detector combinations (i.e., channels).

All fNIRS measurements were performed with a commercial continuous-wave NIR system (NIRScout, NIRx Medical Systems). We designed the optical probe with 16 light sources [light-emitting diodes (LEDs) centered at 760 and 850 nm] and 32 detectors, allowing 64 channels (i.e., source-detector pairs) with distances ranging from 2.8 to 3.5 cm and a temporal resolution of 7.8 Hz. Using a 10–20 standard cap,^53^ the optical probe was placed on the head, covering most of the cortical regions on the temporal, parietal, frontal, and occipital lobes. We used a commercial magnetic motion tracking sensor (Fastrack, Polhemus, Colchester, Vermont) to digitize the location of the optodes on the head and then co-register them on the Colin27 model with the AtlasViewer Matlab package^54^ (Fig. 1). In addition to the optodes, five anatomical landmarks (Nz, Cz, Iz, A1, and A2 in the 10 to 20 system) were also recorded as AtlasViewer requires them to determine the affine transformation from the atlas space to the digitized space of the subject.

For preprocessing the fNIRS data, we used in-house MatLab scripts based on existing HomER 2 functions.^55^ First, we pruned channels with a low SNR (SNR<8; SNR is defined as the mean divided by the standard deviation) and excluded individual runs that did not have at least 50% good channels. Four participants did not have at least four good runs after our pruning criteria and were excluded from further analysis. Finally, we removed consistent bad channels (>70% across all participants) to keep the same number of channels across all subjects. This procedure excluded 16 channels, leading to a probe with 48 remaining channels for further analysis.

For the good-quality channels that survived the quality criteria described above, we converted the measured light intensity to optical density and corrected motion artifacts with a hybrid algorithm that combines spline interpolation with wavelet decomposition, in this order, for properly removing baseline changes and spikes.^9^ With the corrected optical density, we estimated hemoglobin concentration changes using the modified Beer-Lambert law with differential pathlength factor (DPF) equal to 6 for both wavelengths.^56^^,^^57^ The hemoglobin time courses were band-pass filtered between 0.009 and 0.08 Hz to remove high-physiological noise and low-frequency drifts.^20^^,^^22^^,^^48^^,^^49^ Because our goal was to investigate the constraints of subject identification based on neural hemodynamics, we applied a PCA filter to account for the effects of global systemic physiology in fNIRS measurements by removing the first principal component for oxy-hemoglobin (HbO) and deoxy-hemoglobin (HbR).^45^^,^^46^^,^^50^

### 2.4 Resting-State Functional Connectivity Maps

The last preprocessing step removed the temporal autocorrelation from the fNIRS and fMRI time series with an autoregressive model (pre-whitening) of order P to reduce inflated correlations across the brain.^34^^,^^35^^,^^58^^,^^59^ The order of the model was estimated automatically with Bayesian information criteria and independently for each time series of every fNIRS channel or BOLD ROI. We verified that the prewhitening step did not remove the anticorrelation between HbO and HbR (Fig. S1 in the Supplementary Material). For fNIRS, we estimated the total hemoglobin concentration (HbT) changes as the sum of HbO and HbR. Finally, the Pearson correlation coefficients across ROIs (fMRI) and channels (fNIRS) were computed to provide FC matrices for each subject run and brain signal separately (BOLD, HbO, HbR, or HbT).

### 2.5 Subject Identification

The FC matrices (i.e., correlation matrices) were used to identify participants by comparing a subset of the data (known as the testing dataset) with the remaining independently collected runs (referred to as the training dataset). Here, the testing dataset contained one FC matrix per participant (leave-one-out approach), and the training dataset had a variable number of FC matrices with at least one run per participant. We opted to vary the number of samples per subject in the training dataset to investigate how this variation would affect brain fingerprinting’s classification. We evaluated the subject identification performance with either pairwise metrics (see details in Secs. 2.5.1 and 2.5.2) or a linear classifier (see Sec. 2.5.3). For each investigated scenario, the testing and training runs were chosen randomly and independently 300 times. In all cases, the percentage accuracy was defined as

$$\text{Accuracy}(\%)=100\times \frac{\text{Number of corrected labels}}{\text{Number of attempts}}.$$

We used the two-sided Wilcoxon rank-sum test to compare accuracy results obtained under different (independent) scenarios because the distributions in scenarios that yield high accuracy would not be normally distributed. The sample size in all cases was 300 data points. Throughout the results, we provide the Wilcoxon rank sum score for each statistical test and the z-score for computing the approximate p-value of the test. The effect size (estimated with Cohen’s D method) and the p-value adjusted for multiple comparisons (derived from the Bonferroni correction) are also included.

#### 2.5.1 Pearson correlation

In this procedure, subject identification was performed with the Pearson correlation coefficient. This approach, widely used in the literature, assumes that FC matrices from the same participant should have higher correlations among each other than across participants. To this end, we rearranged the upper diagonal elements of each FC matrix (because the FC matrices are symmetric) into a vector and then computed the Pearson correlation coefficients among all possible pairs of testing and training vectors. The participant with the FC matrix that yields the highest correlation with the FC matrix of the testing dataset was assigned as the identified subject.^12^^,^^27^^,^^31^

#### 2.5.2 Geodesic distance

The geodesic distance extends linear metrics, such as the Euclidian distance and the Pearson correlation, by considering that the correlation matrices lie in a nonlinear space.^27^^,^^28^ The geodesic distance between two arbitrary FC matrices ($C_{1}$ and $C_{2}$) was calculated using an affine-invariant Riemannian metric given as

$$\text{Geodesic}(C_{1},C_{2})=\sqrt{\text{trace}[\log^{2}(C_{1}^{-0.5}C_{2}C_{1}^{-0.5})]},$$ (1)

where the log represents the logarithmic matrix operator. To compute the geodesic distance, we added the identity matrix, I, to the FC matrices (i.e., $C_{i}\to C_{i}+I$) to guarantee that all eigenvalues are greater or equal to zero and to avoid convergence problems when inverting the correlation matrices.^27^ With this metric, one assumes that FC matrices from the same participant should have smaller distances to each other than across participants.

#### 2.5.3 Linear classifier

We also examined the performance of a data-driven method for the problem of subject identification. Here, we chose a simple linear classifier without a mapping function (i.e., y^(w,x)=wx, where w and x are the matrix of weights and the attribute vector with incorporated bias, respectively) due to its simplicity.^13^ In this approach, we vectorized the FC matrices as described above and optimized the linear classifier with the training dataset via minimization of the sum of the squares of the residuals with a penalization on the size of the coefficients (ridge regression). The output of the linear classifier was an N-dimensional vector (y^), in which N is the number of participants in the cohort. The j’th element of the output vector, y^j, represents the probability of a given unknown vector from the testing dataset to be identified as subject j. Therefore, the output element with the highest value defined the subject label.

## 3 Results

### 3.1 Number of Runs Available for Training Affects Classification Accuracy

**Fig. 2:**
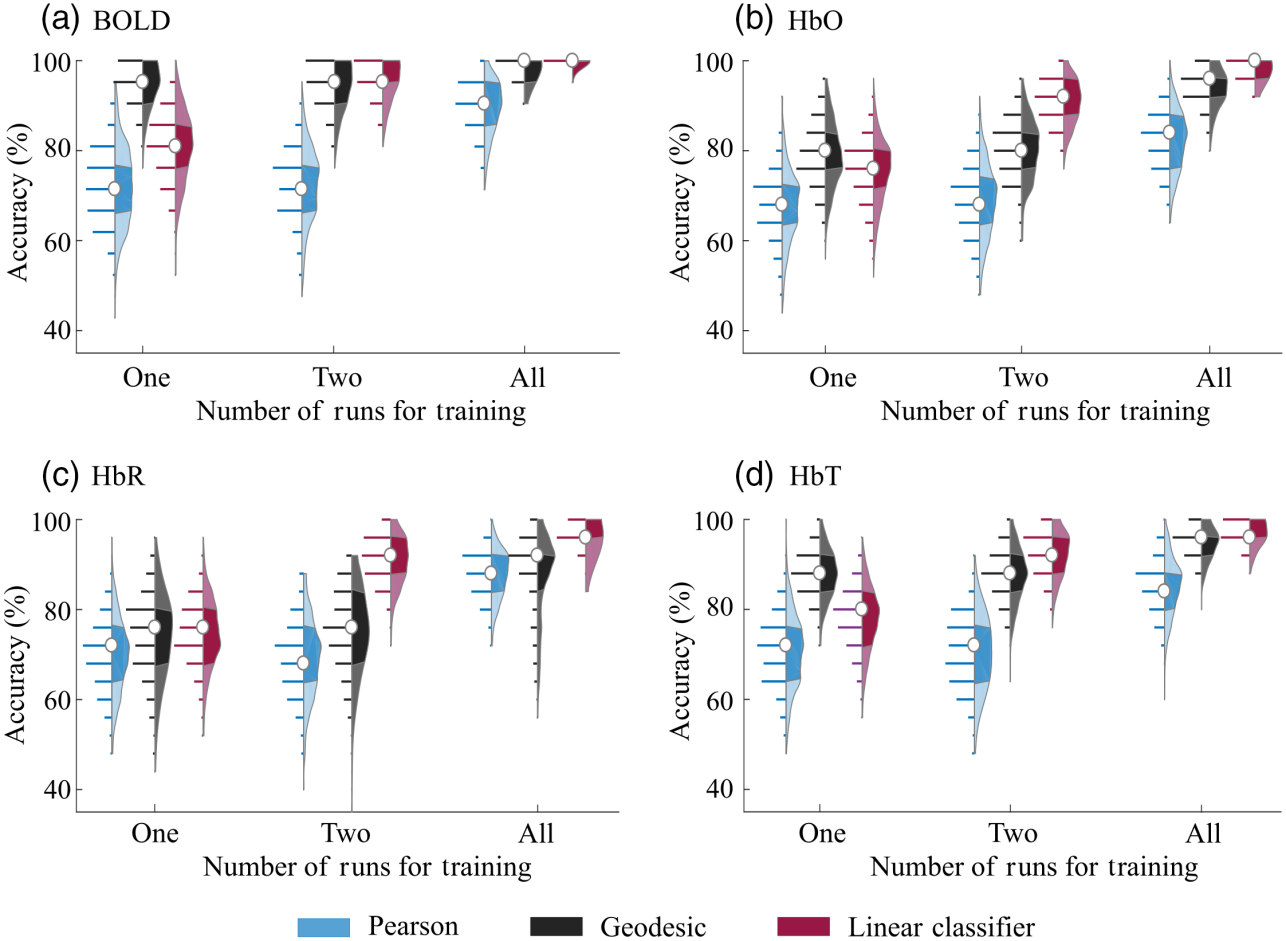
Accuracy of subject identification for Pearson, geodesic, and linear classifier approaches as a function of the number of available runs available for training when (a) BOLD, (b) HbO, (c) HbR, and (d) HbT signals are used separately. The condition “All” refers to the case in which one run was used for testing and the remaining runs were used for training. Due to the pruning criteria, the number of runs per participant varied from three to five for BOLD and four to six for fNIRS. Each distribution depicts all observed results in detail (300 values in total). In each violin plot, the white circle represents the median, and shaded regions indicate the first and third quartiles (as a typical boxplot). The horizontal lines indicate the accuracy values obtained across all trials, and their lengths are proportional to the frequency of each obtained accuracy within the distribution.

First, we investigated how the classification accuracy increases with the number of available runs for training (Fig. 2). For the linear classifier, the average performance increased from 75% to 80% accuracy (when only one run was used for training) to 97% to 99.9% (when we used one run for testing and the remaining runs for training), depending on the contrast used. (Note that, due to the pruning criteria, the number of runs per participant varied from three to five for BOLD and four to six for fNIRS; see Secs. 2.2 and 2.3.) This behavior was also true for the Pearson and geodesic cases, but only when all of the available runs were used for training. In fact, we did not observe relevant differences in accuracy when one or two runs were used for training with these two approaches (Pearson and geodesic), independently of the contrast. As expected, these results illustrate that a minimum amount of temporal information is required to achieve high accuracy; the threshold depends on the contrast provided by the neuroimaging technique and the algorithm used to compare the FC matrices. Overall, at least two 6-min runs per subject in the training dataset appear to be essential for any brain fingerprinting method for fNIRS and BOLD signals.

Concerning the different classification algorithms, the Pearson correlation approach yielded the worst accuracy in all investigated scenarios. Average accuracies barely reached 90%, even when all runs were used for classification. When only one run was available for training, we found that the geodesic distance significantly outperformed the linear classifier by an average (standard deviation) of 11 (6)% for the BOLD, HbO, and HbT contrasts [corrected p-value<0.001 in all cases; BOLD: z-score = 18.35, rank sum score = 128,651, and effect size = 2.05 (1.86 to 2.25); HbO: z-score = 5.53, $\text{rank sum score}=10^{5}$, and effect size = 0.5 (0.34 to 0.66); and HbT: z-score = 15.4, rank sum score = 122,433, and effect size = 1.5 (1.3 to 1.7)]. The only exception was HbR, in which the geodesic distance and the linear classifier presented similar results with a slight advantage to the latter, although not statistically significant [uncorrected p-value = 0.61, z-score = −0.5, rank sum score = 89,084, and effect size = −0.07 (−0.23 to 0.08)].

This situation changed as soon as more information was added to the training set: the linear classifier presented the best performance when more than one run was available for training. The geodesic distance did not change after the addition of a second run, and the average (standard deviation) accuracies of the linear classifier achieved accuracies as high as 95.6 (4.4)%, 90.8 (5.0)%, 90.8 (5.6)%, and 92.3 (5.1)% for the BOLD, HbO, HbR, and HbT signals. On average, this performance was 10% higher than the geodesic distance when the same scenario was considered [corrected p-value<0.001; BOLD: z-score = 3.07, $\text{rank sum score}=9.6\times 10^{4}$, and effect size = 0.29 (0.13 to 0.45); HbO: z-score = 16.5, $\text{rank sum score}=1.2\times 10^{5}$, and effect size = 1.73 (1.55 to 1.92); HbR: z-score = 18.28, $\text{rank sum score}=1.28\times 10^{5}$, and effect size = 1.2 (1.8 to 2.2); ad HbT: z-score = 9.37, $\text{rank sum score}=1.1\times 10^{5}$, and effect size = 0.83 (0.66 to 1.00)]. Together, these results show that the geodesic distance greatly benefits from the nonlinearity when little information is available. However, the heterogeneity captured by adding more independent samples compensates for the nonlinear space if they are all used as different inputs in machine learning algorithms.

Concerning the type of contrast, BOLD provided the highest accuracies compared with any fNIRS signal, and the difference was more remarkable when little information was available (i.e., one run for training). In this scenario, BOLD average accuracy was 1.20, 1.27, and 1.08 times greater than HbO, HbR, and HbT, respectively. In fact, BOLD was the only contrast to achieve 90% accuracy when only one run was available for training. Considering the best approach per training condition and brain signal, we observed that the BOLD classifications were still the highest. However, the discrepancy in accuracy between BOLD and fNIRS decreased as the number of runs used for training increased. In the condition with the most runs available for training, the average accuracy of BOLD/fNIRS was 1.09 (HbO), 1.12 (HbR), and 1.05 (HbT).

### 3.2 Amount of Spatial Information Influences Performance on Subject Identification

The minimum amount of temporal information for achieving high accuracy may vary with the spatial information available for analysis. In fact, the greater spatial heterogeneity captured by the higher number of ROIs in BOLD (94 regions, creating a 94×94 FC matrix) appears to be the main reason for its higher accuracy when compared with fNIRS (48 channels, creating a 48×48 FC matrix).

To investigate whether the larger spatial information drove the higher BOLD accuracy, we evaluated the classification performances as a function of the number of available ROIs. For each modality, we selected random sub-networks with 10, 20, 30, 40, and 48 ROIs and performed the classifications with one run for training and one for testing. The chosen ROIs were randomly selected and repeated ten times (except for the case of using all ROIs), avoiding discrepant accuracy values due to a given joint of ROIs. For each subgroup of ROIs, the set of runs for training and testing was randomly selected and repeated 300 times, as done in the other comparisons. We focused on the geodesic distance because it provided the highest performance when only one run was available for training.

**Fig. 3:**
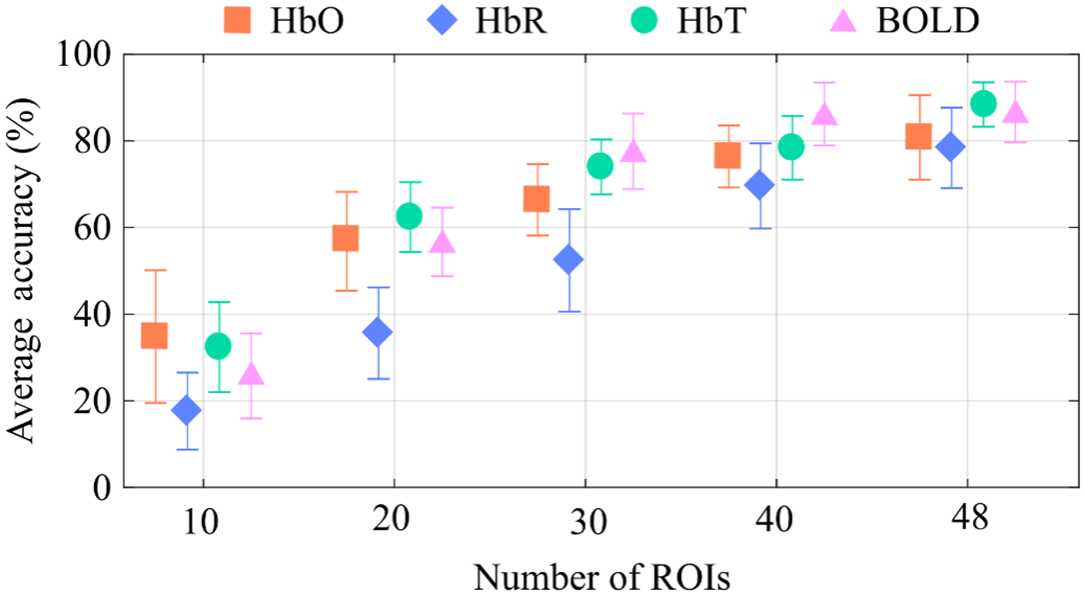
Average geodesic classification accuracy as a function of the number of ROIs when only one run was used for training and another for testing. The error bars represent the standard deviation. The performance on subject identification increases with the number of ROIs used in the classification for all contrasts (BOLD, HbO, HbR, and HbT); in particular, HbT accuracy performed similarly to BOLD when the number of ROIs across the two techniques was equal.

Figure 3 shows how the average accuracy increases with the number of ROIs used as inputs in the classification, confirming that a broader coverage of independent ROIs contributes to extracting unique and reliable connectivity patterns for subject identification. (Figure S2 in the Supplementary Material depicts the whole distribution.) Interestingly, the BOLD-based accuracy did not differ from the HbT accuracy when the two techniques matched the same number of ROIs. This result suggests that the higher performance from BOLD in Fig. 2 likely comes from the higher number of ROIs sampled.

Unlike fMRI, the brain regions measured with fNIRS depend on the instrument setup—specifically, the number of sources and detectors and the density of channels created for a given region. (Note that the number of ROIs may not be directly associated with the number of channels. For example, high-density fNIRS probes with all channels placed locally sample a small ROI, and the information provided by this probe will have a high covariance across channels.) Therefore, the spatial information is highly variable across different fNIRS studies. We attempted to estimate the relationship between the classification accuracy and the number of independent regions measured. Specifically, we approximated the average accuracy to an empirical model that resembles the behavior observed for all contrasts in Fig. 3, i.e., $\text{Accuracy}(\%)=\alpha \times (1-e^{-\gamma (\text{Number of ROIs})})$. Table 1 shows the estimated parameters and the chi-square ($\chi^{2}$) goodness-of-fit test (under the null hypothesis that the data follows the specified model). One interesting prediction from the model is that, although HbO and HbT are typically reported in resting-state fNIRS studies, the information contained in these contrasts is limited, with a predicted saturation (estimated by α in our model) of 88% and 98%, respectively. In other words, our model suggests that these contrasts would not provide a target of 100% accuracy with just one single run for training, even in the limit of hundreds of fNIRS channels.

**Table 1:** Estimates for the data shown in Fig. 3 with the model predicting the accuracy as a function of the number of ROIs. Chi-square estimate ($\chi^{2}$) shows the goodness-of-fit under the null hypothesis that the data follows the specified model. In our problem, the critical $\chi^{2}$ to reach a significance level of 0.01 must be less than or equal to 0.115 (3 degrees of freedom). The scores ${\mathrm{AR}}_{0.5\%}/{\mathrm{AR}}_{0.25\%}$ represent the threshold at which adding an extra ROI would increase the accuracy by <0.5%/0.25%, respectively.

Neuroimaging contrast	α	γ	$\chi^{2}$	${\mathrm{AR}}_{0.5\%}$	${\mathrm{AR}}_{0.25\%}$
**HbO**	88	0.05	$9\times 10^{-4}$	44	58
**HbR**	100	0.027	$3\times 10^{-2}$	63	89
**HbT**	98	0.046	$8\times 10^{-3}$	48	63
**BOLD**	100	0.043	$3\times 10^{-2}$	50	67

By taking the derivative of the above expression for accuracy with respect to the number of ROIs, we can also estimate the improvement rate in the classification accuracy (AR) as a function of the spatial information. As the accuracy increases with the number of ROIs, the improvement rate will decrease exponentially by a factor γ in our model. Table 1 shows two relevant AR thresholds: 0.50% (${\mathrm{AR}}_{0.5\%}$) and 0.25% (${\mathrm{AR}}_{0.25\%}$). The number of ROIs in which the improvement in the accuracy rate is lower than 0.50% (${\mathrm{AR}}_{0.5\%}$) can be interpreted as the minimum number of channels for achieving good classification (because there would be a huge margin for significant improvement in accuracy with fewer channels), whereas ${\mathrm{AR}}_{0.25\%}$ can be seen as a “saturating” point for accuracy, i.e., at this point, increasing the number of channels does not considerably increase the accuracy performance. With our data, the number of necessary ROIs to achieve ${\mathrm{AR}}_{0.5\%}$ and ${\mathrm{AR}}_{0.25\%}$ was the highest for HbR when compared with HbO and HbT. This result suggests that the HbR-based resting-state FC (rsFC) needs a broader probe coverage to extract unique individual features to identify participants with the same precision as HbO, HbT, and BOLD. In addition, the model estimates for HbT are comparable to BOLD, reinforcing that this fNIRS parameter could be more suitable to replicating BOLD-based resting-state FC results, as empirically suggested by previous studies.^20^^,^^22^^,^^45^^,^^60^^,^^61^

### 3.3 HbR Provides Additional Complementary Information to HbT FC Maps

**Fig. 4:**
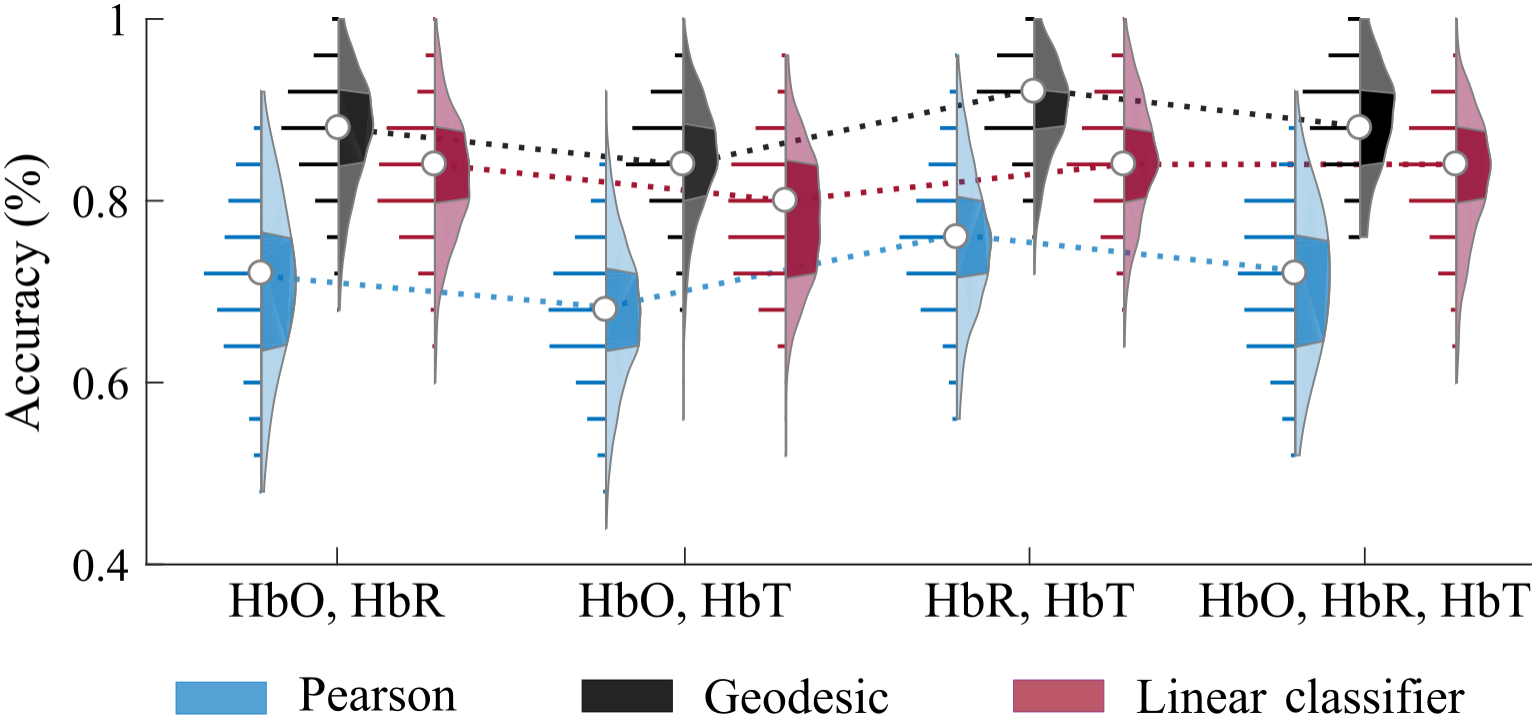
Classification accuracy during subject identification when combining fNIRS contrasts. The accuracy was calculated using the Pearson, geodesic, and linear classifier approaches. We used only one run for training and another for testing in this case. Combining HbR and HbT presented the highest performance across all possible combinations. Each distribution depicts all observed results in detail (300 values in total). In each violin plot, the white circle represents the median, and shaded regions indicate the first and third quartiles (as a typical boxplot). The horizontal lines show the observed accuracy values, and their lengths are proportional to the frequency of each finding inside the distribution. The dotted lines are provided to facilitate comparison across the different combinations only.

Finally, we explored how combining different fNIRS chromophores would benefit subject identification (Fig. 4). For the Pearson correlation and linear classifier approaches, we combined different hemoglobin information by concatenating their vectorized correlation matrices. For the geodesic distance, we concatenated the FC matrices through the main diagonal to obtain block-diagonal matrices and preserve the spatial properties of the original FC matrices.^28^ For this investigation, we used only one run for training and one run for testing because our goal was to compare the combination of contrasts with the effects of having more runs for training.

Overall, combining fNIRS contrasts improves subject identification, suggesting that the different hemoglobin contrasts carry complementary information for extracting connectivity patterns at the individual level. However, it does not compensate for more data points (i.e., having more runs for training is still better than just combining fNIRS data). Figure 4 shows the average accuracy for all possible hemoglobin combinations (see Fig. S3 in the Supplementary Material for the confusion matrices). Concerning all different possibilities, the combination of HbR and HbT provided the highest performance, with an average (standard deviation) accuracy of 90.2 (5.2)%—which is comparable to the 92.3 (5.1)% average accuracy that we obtained for HbT-only with two runs for training. Interestingly, adding HbO to the HbR+HbT combination did not increase accuracy. Concerning the different classification strategies, the geodesic distance again outperformed the other approaches in all cases.

## 4 Discussion

This work focuses on identifying subjects based on their neural patterns as measured by rsFC, so called brain fingerprinting. Although brain fingerprinting has not gained practical application, it illustrates the value of improving data acquisition and analysis methods to enhance intra-subject reproducibility with neuroimaging techniques. We believe that the search for individual features should be further explored in functional neuroimaging despite the scientific challenges because accurate functional cortical information holds the potential to guide treatment or rehabilitation decisions, favoring patient recovery and outcome in the short term.^30^ As subject identification is likely to be more dependent on acquisition conditions than analysis pipelines, we employed a validated analysis pipeline that was previously demonstrated to reproduce resting-state networks commonly reported in the literature^45^ and examined how spatiotemporal properties affect reproducibility and, thus, brain fingerprinting.

It is quite straightforward—and even intuitive—that data acquisition conditions will influence the ability to identify subjects. For each subject, it is ideal to collect several runs from many different ROIs for as long as possible with a variety of techniques to obtain as much information as possible. However, this approach is neither feasible nor scalable in practice. The fNIRS systems have a limited number of channels to probe different ROIs that depend on the number of sources and detectors and how dense the coverage of a single region should be. In addition, collecting long datasets from multiple runs or sessions is time-consuming, demands a high operational cost, and is restricted to specific populations. Hence, real datasets have tradeoffs, and the present work investigates relevant acquisition constraints and limitations associated with the different choices that must be made for solving the problem of brain fingerprinting using resting-state functional measurements.

Overall, our results show that FC maps obtained at rest carry sufficient information to identify participants with near-perfect accuracy. This finding indicates that either fNIRS or fMRI can isolate the uniqueness of individual brains despite the presence of robust inter-subject resting-state networks.^45^^,^^62^^,^^63^ Our ability to identify distinctive and reliable brain features across such a homogenous group of mainly young males (26 out of 29), which presents a significant challenge for the subject classification methodologies, needs to be highlighted. In samples with an even sex balance, sex-related differences could facilitate subject identification because FC is known to differ according to sex.^64^

Previous fMRI studies have introduced different approaches for performing subject identification, primarily based on pairwise distance metrics, such as the Pearson correlation and geodesic distance.^27^ In this vein, our results reinforce the better performance of the geodesic distance over the Pearson correlation. We additionally explored how standard machine learning algorithms contribute to brain fingerprinting. Here, we chose to use a linear classifier without a mapping function. Our results show that this algorithm was sufficient for capturing the individual variability when enough data were available, suggesting that the choice of data-driven algorithms is not the main factor that drives this problem; consequently, using more computationally intensive algorithms does not add any significant contributions to brain fingerprinting under these conditions. In fact, we tested the performance of a standard multilayer perceptron (MLP) artificial neural network in our data under different conditions (results not shown). Specifically, we tested several MLP architectures by varying the number of hidden layers (1, 2, and 3) and the number of neurons per hidden layer. In all investigated scenarios, the MLP did not yield higher accuracies than the linear classifier. From the computational perspective, the better performance of the linear classifier is likely related to the relatively low amount of data available per subject (in this study, five runs for training in the best case).

However, very few experimental protocols acquire more temporal data than ours; therefore, the main advantages of conventional machine learning approaches will be limited in functional neuroimaging data given the current experimental protocols (although they would truly benefit from larger databases, such as the human connectome project available for fMRI,^65^ for such datasets are still not available for fNIRS). The use of few-shot learning algorithms, in which a machine learning model learns from only one or a few samples, would probably be more beneficial to fNIRS than conventional deep-learning neural networks.^66^ In addition, another promising alternative would be to add a kernel to the linear classifier or employ a nearest neighbors’ algorithm (NNA). A proper kernelization strategy could improve the performance of the linear classifier to excel the geodesic distance, and the NNA should perform at least better than the Pearson correlation. Meanwhile, acquiring more data from broader ROIs would bring more value to the goal of reducing intra-subject variability for single-subject analysis.

A relevant methodological difference between pairwise metrics and machine learning approaches is how they use the temporal information available. When more than one run is available, the correlation matrices are typically averaged across runs in the Pearson and geodesic distance calculation.^12^^,^^27^ For the linear classifier, each FC matrix is used as an independent sample for each participant.^13^ Using every run as a sample will be more robust than averages because it will not average out dynamic features that are not present in every run but still characterize each participant.^67^^–^^69^ For this reason, the linear approach outperformed the other methods for the fNIRS signals when more than one run was available for training (Fig. 2). The difference between the linear classifier and the geodesic distance is not as evident for fMRI as for the fNIRS because the correct computation of the pairwise distance in the nonlinear correlation space across a broad spatial dataset compensates for the lost temporal information. Thus, it is possible that the geodesic distance could have a similar performance to the linear classifier when used on broad fNIRS probes that cover independent regions, as suggested by the results presented in Fig. 3. In the case in which more spatial information is available, the geodesic distance would probably be preferred over machine learning techniques due to its simplicity and low computational cost.

The number of covered ROIs (not necessarily the number of fNIRS channels or fMRI voxels) is a crucial factor in extracting specific and meaningful information to identify individuals. The behavior seen in Fig. 3 indicates that measuring more brain regions can uncover unique connectivity patterns and favor subject identification. Because fMRI experiments naturally probe the entire brain cortex, this finding is more relevant to the fNIRS field; it implies that, although challenging, whole-head fNIRS measurements are recommended for individual monitoring and are preferred over dense local fNIRS measurements if sufficient sources and detectors are not available.

In terms of identifying subjects, it would be interesting to isolate which brain areas, if any, are most relevant in the future. Although we did not isolate which subnetworks could play a more important role to subject differentiation, it has been argued that frontoparietal networks are crucial to individual uniqueness.^12^ Frontoparietal networks are more linked to higher-order cognitive processing than primary sensory functions; thereby, it is not unreasonable to expect that these networks may encode the uniqueness of each brain. Interestingly, frontoparietal networks have also demonstrated great value in the clinic at the individual level for quantifying residual cognitive function in patients with disorders of consciousness.^70^^,^^71^ In the future, combining the knowledge of the relevant brain regions with real-time neuronavigation for accurate fNIRS placement of sparse optical probes could overcome constraints due to a limited number of optodes and avoid unnecessary broad optical designs that are often time-consuming to set up.^10^^,^^24^

Among all fNIRS contrasts, HbT provided the highest classification accuracies, which is consistent with previous studies that found HbT to be the most reliable feature for rsFC.^20^^,^^22^^,^^45^^,^^60^ By combining HbO and HbR, HbT captures information from both. However, one might hypothesize that combining different fNIRS contrasts would still yield complementary information due to differences in HbO, HbR, and HbT dynamics during brain activity.^5^^,^^38^^,^^72^^–^^74^ In fact, adding HbR to HbT resulted in improved classification, but adding HbO to the combination of HbR and HbT made no difference in average accuracy (Fig. 4). As HbO fluctuations are higher than HbR fluctuations, they drive HbT fluctuations, which may explain why HbO does not increase accuracy in the presence of HbT. In contrast, HbR contributes little to HbT, so combining the two will give HbR more weight in the classification problem, which can help identify subjects.

We found, however, that the combination of fNIRS contrasts only showed modest improvements in classification accuracy overall and did not compensate for the acquisition of multiple runs from the same subject. In addition, the combined data did not reach accuracies close to the ones obtained with the BOLD signal, indicating that the spatial information is also more relevant than the complementary dynamics of HbO, HbR, and HbT. The low improvement from the combination of fNIRS contrasts is probably related to the high cross-correlation among HbO, HbR, and HbT; note that, for neural hemodynamics, HbO and HbR measured in the same channel are expected to be anticorrelated. Of relevant note on this topic, in this work, we combined the FC matrices from each hemoglobin by concatenating their correlation values. An alternative approach would be to treat each fNIRS signal as independent input and then compute the Pearson correlation coefficient across them. Therefore, we would have access to the cross-correlations between HbO, HbR, and HbT instead of only their concatenated correlation values. This approach, combined with the geodesic distance, could potentially increase the observed rates.

For the sole purpose of subject identification, it is worth noting that the fNIRS sensitivity to extracerebral hemodynamics and systemic physiology could also be used as an additional feature (although it would not constitute brain fingerprinting). In fact, for this particular study, in which data were obtained within a few minutes of each other during the same session, the additional information provided by global hemodynamics (which we removed with PCA) could facilitate the identification of subjects from fNIRS data and increase the classification accuracies reported above. However, systemic hemodynamics inherent in fNIRS data can be difficult to generalize to a more global context. Using only systemic information might not be beneficial to subject identification because global systemic hemodynamics are governed by feedback and feedforward mechanisms of the nervous system as a response to the body’s needs. For a given subject, the systemic pattern would likely be more variable than the neural pattern if data were acquired at different sessions.

## 5 Conclusion

In summary, we investigated the problem of subject identification with resting-state brain FC through simultaneous measurements with fMRI and fNIRS data from 29 healthy and young subjects. To our knowledge, no previous work has performed a comprehensive study of the feasibility of using fNIRS for identifying participants during the resting state with fMRI validation. Considering the results obtained with our dataset, we suggest acquiring at least four runs per subject (three for training) to achieve close to 100% accuracy. For the fNIRS measurements, it is important to have a wide coverage with ∼50 channels (ROIs). In addition, the combination of HbR and HbT provides higher classification performance than each hemoglobin separately. We highlight that correctly classifying subjects in such a homogenous group as ours shows the tremendous potential of fMRI and fNIRS for intra-subject analysis, particularly with rsFC.

## Data Availability

Data and code underlying the results presented in this paper may be obtained from the authors upon reasonable request.

## References

[1] Huppert T. J. et al., “Quantitative spatial comparison of diffuse optical imaging with blood oxygen level-dependent and arterial spin labeling-based functional magnetic resonance imaging,” J. Biomed. Opt. 11(6), 064018 (2006). JBOPFO 1083-3668 10.1117/1.2400910 17212541 PMC2670188

[r2] Huppert T. J. et al., “A temporal comparison of BOLD, ASL, and NIRS hemodynamic responses to motor stimuli in adult humans,” Neuroimage 29(2), 368–382 (2006). NEIMEF 1053-8119 10.1016/j.neuroimage.2005.08.065 16303317 PMC2692693

[r3] Steinbrink J. et al., “Illuminating the BOLD signal: combined fMRI–fNIRS studies,” Magn. Reson. Imaging 24(4), 495–505 (2006). MRIMDQ 0730-725X 10.1016/j.mri.2005.12.034 16677956

[r4] Schaeffer S. Iadecola C., “Revisiting the neurovascular unit,” Nat. Neurosci. 24(9), 1198–1209 (2021). NANEFN 1097-6256 10.1038/s41593-021-00904-7 34354283 PMC9462551

[5] Mesquita R. C. Huppert T. J. Boas D. A., “Exploring neuro-vascular and neuro-metabolic coupling in rat somatosensory cortex,” Phys. Med. Biol. 54(2), 175–185 (2009). PHMBA7 0031-9155 10.1088/0031-9155/54/2/001 19088392 PMC2637347

[6] Chen J. E. et al., “Resting-state ‘physiological networks,’” Neuroimage 213(February), 116707 (2020). NEIMEF 1053-8119 10.1016/j.neuroimage.2020.116707 32145437 PMC7165049

[r7] Tong Y. et al., “Evaluating the effects of systemic low frequency oscillations measured in the periphery on the independent component analysis results of resting state networks,” Neuroimage 76, 202–215 (2013). NEIMEF 1053-8119 10.1016/j.neuroimage.2013.03.019 23523805 PMC3652630

[r8] Kirilina E. et al., “Identifying and quantifying main components of physiological noise in functional near infrared spectroscopy on the prefrontal cortex,” Front. Hum. Neurosci. 7, 864 (2013). 10.3389/fnhum.2013.00864 24399947 PMC3865602

[r9] Novi S. L. et al., “Functional near-infrared spectroscopy for speech protocols: characterization of motion artifacts and guidelines for improving data analysis,” Neurophotonics 7(1), 1 (2020). 10.1117/1.NPh.7.1.015001 PMC6953699 31956662

[r10] Novi S. L. et al., “Integration of spatial information increases reproducibility in functional near-infrared spectroscopy,” Front. Neurosci. 14, 746 (2020).1662-453X 10.3389/fnins.2020.00746 32848543 PMC7399018

[11] Abdalmalak A. et al., “Effects of systemic physiology on mapping resting-state networks using functional near-infrared spectroscopy,” Front. Neurosci. 16(March), 12 (2022).1662-453X 10.3389/fnins.2022.803297 PMC8957952 35350556

[12] Finn E. S. et al., “Functional connectome fingerprinting: identifying individuals using patterns of brain connectivity,” Nat. Neurosci. 18(11), 1664–1671 (2015). NANEFN 1097-6256 10.1038/nn.4135 26457551 PMC5008686

[r13] Arbabshirani M. R. et al., “Single subject prediction of brain disorders in neuroimaging: promises and pitfalls,” Neuroimage 145, 137–165 (2017). NEIMEF 1053-8119 10.1016/j.neuroimage.2016.02.079 27012503 PMC5031516

[14] Amico E. Goñi J., “The quest for identifiability in human functional connectomes,” Sci. Rep. 8(1), 8254 (2018). SRCEC3 2045-2322 10.1038/s41598-018-25089-1 29844466 PMC5973945

[15] Niu H. et al., “Test-retest reliability of graph metrics in functional brain networks: a resting-state fNIRS study,” PLoS One 8(9), e72425 (2013). POLNCL 1932-6203 10.1371/journal.pone.0072425 24039763 PMC3767699

[r16] Braun U. et al., “Test-retest reliability of resting-state connectivity network characteristics using fMRI and graph theoretical measures,” Neuroimage 59(2), 1404–1412 (2012). NEIMEF 1053-8119 10.1016/j.neuroimage.2011.08.044 21888983

[r17] Rombouts S. A. R. B. et al., “Test-retest analysis with functional MR of the activated area in the human visual cortex,” Am. J. Neuroradiol. 18(7), 1317–1322 (1997). 9282862 PMC8338037

[r18] Kono T. et al., “Multiple-time replicability of near-infrared spectroscopy recording during prefrontal activation task in healthy men,” Neurosci. Res. 57(4), 504–512 (2007). 10.1016/j.neures.2006.12.007 17250915

[r19] Blasi A. et al., “Test–retest reliability of functional near infrared spectroscopy in infants,” Neurophotonics 1(2), 025005 (2014). 10.1117/1.NPh.1.2.025005 26157978 PMC4478781

[r20] Mesquita R. C. Franceschini M. A. Boas D. A., “Resting state functional connectivity of the whole head with near infrared spectroscopy,” Biomed. Opt. Express 1(1), 324–336 (2010). BOEICL 2156-7085 10.1364/BOE.1.000324 21258470 PMC3005169

[r21] Noble S. et al., “Multisite reliability of MR-based functional connectivity,” Neuroimage 146, 959–970 (2017). NEIMEF 1053-8119 10.1016/j.neuroimage.2016.10.020 27746386 PMC5322153

[22] Novi S. L. Rodrigues R. B. M. L. Mesquita R. C., “Resting state connectivity patterns with near-infrared spectroscopy data of the whole head,” Biomed. Opt. Express 7(7), 2524 (2016). BOEICL 2156-7085 10.1364/BOE.7.002524 27446687 PMC4948611

[r23] Power J. D. et al., “Spurious but systematic correlations in functional connectivity MRI networks arise from subject motion,” Neuroimage 59(3), 2142–2154 (2012). NEIMEF 1053-8119 10.1016/j.neuroimage.2011.10.018 22019881 PMC3254728

[r24] Wu S.-T. et al., “Accurate image-guided (re)placement of NIRS probes,” Comput. Methods Programs Biomed. 200, 105844 (2021). CMPBEK 0169-2607 10.1016/j.cmpb.2020.105844 33267972

[r25] Fox M. D. Raichle M. E., “Spontaneous fluctuations in brain activity observed with functional magnetic resonance imaging,” Nat. Rev. Neurosci. 8(9), 700–711 (2007). NRNAAN 1471-003X 10.1038/nrn2201 17704812

[26] Power J. D. et al., “Methods to detect, characterize, and remove motion artifact in resting state fMRI,” Neuroimage 84, 320–341 (2014). NEIMEF 1053-8119 10.1016/j.neuroimage.2013.08.048 23994314 PMC3849338

[27] Venkatesh M. Jaja J. Pessoa L., “Comparing functional connectivity matrices: a geometry-aware approach applied to participant identification,” Neuroimage 207, 116398 (2020). NEIMEF 1053-8119 10.1016/j.neuroimage.2019.116398 31783117 PMC6995445

[28] Pennec X. Fillard P. Ayache N., “A Riemannian framework for tensor computing,” Int. J. Comput. Vis. 66(1), 41–66 (2006). IJCVEQ 0920-5691 10.1007/s11263-005-3222-z

[29] Kazazian K. et al., “Improving diagnosis and prognosis in acute severe brain injury: a multimodal imaging protocol,” Front. Neurol. 12, 757219 (2021). 10.3389/fneur.2021.757219 34938260 PMC8685572

[30] Thair H. et al., “Transcranial direct current stimulation (tDCS): a beginner’s guide for design and implementation,” Front. Neurosci. 11(Nov), 1–13 (2017).1662-453X 10.3389/fnins.2017.00641 29213226 PMC5702643

[31] de Souza Rodrigues J. et al., “Identifying individuals using fNIRS-based cortical connectomes,” Biomed. Opt. Express 10(6), 2889 (2019). BOEICL 2156-7085 10.1364/BOE.10.002889 31259059 PMC6583329

[32] Ren H. et al., “Identifying individuals by fNIRS-based brain functional network fingerprints,” Front. Neurosci. 16, 813293 (2022).1662-453X 10.3389/fnins.2022.813293 35221902 PMC8873366

[33] Huppert T. J., “Commentary on the statistical properties of noise and its implication on general linear models in functional near-infrared spectroscopy,” Neurophotonics 3(1), 010401 (2016). 10.1117/1.NPh.3.1.010401 26989756 PMC4773699

[34] Arbabshirani M. R. et al., “Impact of autocorrelation on functional connectivity,” Neuroimage 102(P2), 294–308 (2014). NEIMEF 1053-8119 10.1016/j.neuroimage.2014.07.045 25072392 PMC4253536

[35] Santosa H. et al., “Characterization and correction of the false-discovery rates in resting state connectivity using functional near-infrared spectroscopy,” J. Biomed. Opt. 22(5), 055002 (2017). JBOPFO 1083-3668 10.1117/1.JBO.22.5.055002 28492852 PMC5424771

[36] Saager R. B. Berger A. J., “Direct characterization and removal of interfering absorption trends in two-layer turbid media,” J. Opt. Soc. Am. A 22(9), 1874 (2005). JOAOD6 0740-3232 10.1364/JOSAA.22.001874 16211814

[r37] Brigadoi S. et al., “Motion artifacts in functional near-infrared spectroscopy: a comparison of motion correction techniques applied to real cognitive data,” Neuroimage 85, 181–191 (2014). NEIMEF 1053-8119 10.1016/j.neuroimage.2013.04.082 23639260 PMC3762942

[r38] Yücel M. A. et al., “Best practices for fNIRS publications,” Neurophotonics 8(1), 012101 (2021). 10.1117/1.NPh.8.1.012101 33442557 PMC7793571

[r39] Yücel M. A. et al., “Short separation regression improves statistical significance and better localizes the hemodynamic response obtained by near-infrared spectroscopy for tasks with differing autonomic responses,” Neurophotonics 2(3), 035005 (2015). 10.1117/1.NPh.2.3.035005 26835480 PMC4717232

[r40] Gagnon L. et al., “Short separation channel location impacts the performance of short channel regression in NIRS,” Neuroimage 59(3), 2518–2528 (2012). NEIMEF 1053-8119 10.1016/j.neuroimage.2011.08.095 21945793 PMC3254723

[r41] Brigadoi S. Cooper R. J., “How short is short? Optimum source–detector distance for short-separation channels in functional near-infrared spectroscopy,” Neurophotonics 2(2), 025005 (2015). 10.1117/1.NPh.2.2.025005 26158009 PMC4478880

[r42] Mesquita R. C. et al., “Influence of probe pressure on the diffuse correlation spectroscopy blood flow signal: extra-cerebral contributions,” Biomed. Opt. Express 4(7), 978 (2013). BOEICL 2156-7085 10.1364/BOE.4.000978 23847725 PMC3704102

[r43] Caldwell M. et al., “Modelling confounding effects from extracerebral contamination and systemic factors on functional near-infrared spectroscopy,” Neuroimage 143, 91–105 (2016). NEIMEF 1053-8119 10.1016/j.neuroimage.2016.08.058 27591921 PMC5139986

[r44] Zhang H. et al., “Test-retest assessment of independent component analysis-derived resting-state functional connectivity based on functional near-infrared spectroscopy,” Neuroimage 55(2), 607–615 (2011). NEIMEF 1053-8119 10.1016/j.neuroimage.2010.12.007 21146616

[45] Abdalmalak A. et al., “Effects of systemic physiology on mapping resting-state networks using functional near-infrared spectroscopy,” Front. Neurosci. 16, 803297 (2022).1662-453X 10.3389/fnins.2022.803297 35350556 PMC8957952

[46] Zhang Y. et al., “Eigenvector-based spatial filtering for reduction of physiological interference in diffuse optical imaging,” J. Biomed. Opt. 10(1), 011014 (2005). JBOPFO 1083-3668 10.1117/1.1852552 15847580

[47] de Campos B. M. Casseb R. F. Cendes F., “UF2C — user-friendly functional connectivity: a neuroimaging toolbox for fMRI processing and analyses,” SoftwareX 11, 100434 (2020). 10.1016/j.softx.2020.100434

[48] Sasai S. et al., “A NIRS–fMRI study of resting state network,” Neuroimage 63(1), 179–193 (2012). NEIMEF 1053-8119 10.1016/j.neuroimage.2012.06.011 22713670

[r49] Sasai S. et al., “Frequency-specific functional connectivity in the brain during resting state revealed by NIRS,” Neuroimage 56(1), 252–257 (2011). NEIMEF 1053-8119 10.1016/j.neuroimage.2010.12.075 21211570

[50] Carbonell F. Bellec P. Shmuel A., “Global and system-specific resting-state fMRI fluctuations are uncorrelated: principal component analysis reveals anti-correlated networks,” Brain Connect. 1(6), 496–510 (2011). 10.1089/brain.2011.0065 22444074 PMC3604782

[51] Van Dijk K. R. A. et al., “Intrinsic functional connectivity as a tool for human connectomics: theory, properties, and optimization,” J. Neurophysiol. 103(1), 297–321 (2010). JONEA4 0022-3077 10.1152/jn.00783.2009 19889849 PMC2807224

[52] Rolls E. T. Joliot M. Tzourio-Mazoyer N., “Implementation of a new parcellation of the orbitofrontal cortex in the automated anatomical labeling atlas,” Neuroimage 122, 1–5 (2015). NEIMEF 1053-8119 10.1016/j.neuroimage.2015.07.075 26241684

[53] Klem G. H. et al., “The ten twenty electrode system: international federation of societies for electroencephalography and clinical neurophysiology,” Electroencephalogr. Clin. Neurophysiol. 10(2), 370–375 (1958). ECNEAZ 0013-4694 10.1016/0013-4694(58)90053-1 10590970

[54] Aasted C. M. et al., “Anatomical guidance for functional near-infrared spectroscopy: AtlasViewer tutorial,” Neurophotonics 2(2), 020801 (2015). 10.1117/1.NPh.2.2.020801 26157991 PMC4478785

[55] Huppert T. J. et al., “HomER: a review of time-series analysis methods for near-infrared spectroscopy of the brain,” Appl. Opt. 48(10), 280–298 (2009). APOPAI 0003-6935 10.1364/AO.48.00D280 PMC2761652 19340120

[56] Delpy D. et al., “Estimation of optical pathlength through tissue from direct time of flight measurements,” Phys. Med. Biol. 33(12), 1433–1442 (1988). PHMBA7 0031-9155 10.1088/0031-9155/33/12/008 3237772

[57] Franceschini M. A. et al., “Diffuse optical imaging of the whole head,” J. Biomed. Opt. 11(5), 054007 (2006). JBOPFO 1083-3668 10.1117/1.2363365 17092156 PMC2637816

[58] Barker J. W. Aarabi A. Huppert T. J., “Autoregressive model based algorithm for correcting motion and serially correlated errors in fNIRS,” Biomed. Opt. Express 4(8), 1366 (2013). BOEICL 2156-7085 10.1364/BOE.4.001366 24009999 PMC3756568

[59] Blanco B. Molnar M. Caballero-Gaudes C., “Effect of prewhitening in resting-state functional near-infrared spectroscopy data,” Neurophotonics 5(4), 040401 (2018). 10.1117/1.NPh.5.4.040401 30397629 PMC6200149

[60] Culver J. P. et al., “Evidence that cerebral blood volume can provide brain activation maps with better spatial resolution than deoxygenated hemoglobin,” Neuroimage 27(4), 947–959 (2005). NEIMEF 1053-8119 10.1016/j.neuroimage.2005.05.052 16084112

[61] Sheth S. A., “Columnar specificity of microvascular oxygenation and volume responses: implications for functional brain mapping,” J. Neurosci. 24(3), 634–641 (2004). JNRSDS 0270-6474 10.1523/JNEUROSCI.4526-03.2004 14736849 PMC6729264

[62] van den Heuvel M. P. Hulshoff Pol H. E., “Exploring the brain network: a review on resting-state fMRI functional connectivity,” Eur. Neuropsychopharmacol. 20(8), 519–534 (2010). EURNE8 0924-977X 10.1016/j.euroneuro.2010.03.008 20471808

[63] van Dijk K. R. A. et al., “Intrinsic functional connectivity as a tool for human connectomics: theory, properties, and optimization,” J. Neurophysiol. 103(1), 297–321 (2010). JONEA4 0022-3077 10.1152/jn.00783.2009 19889849 PMC2807224

[64] Weis S. et al., “Sex classification by resting state brain connectivity,” Cereb. Cortex 30(2), 824–835 (2020). 53OPAV 1047-3211 10.1093/cercor/bhz129 31251328 PMC7444737

[65] van Essen D. C. et al., “The WU-Minn human connectome project: an overview,” Neuroimage 80, 62–79 (2013). NEIMEF 1053-8119 10.1016/j.neuroimage.2013.05.041 23684880 PMC3724347

[66] Sucholutsky I. Schonlau M., “‘Less than one’-shot learning: learning N classes from M < N samples” (2021).

[67] Abrol A. et al., “Replicability of time-varying connectivity patterns in large resting state fMRI samples,” Neuroimage 163(September), 160–176 (2017). NEIMEF 1053-8119 10.1016/j.neuroimage.2017.09.020 28916181 PMC5775892

[r68] Allen E. A. et al., “Capturing inter-subject variability with group independent component analysis of fMRI data: a simulation study,” Neuroimage 59(4), 4141–4159 (2012). NEIMEF 1053-8119 10.1016/j.neuroimage.2011.10.010 22019879 PMC3690335

[69] Allen E. A. et al., “Tracking whole-brain connectivity dynamics in the resting state,” Cereb. Cortex 24(3), 663–676 (2014). 53OPAV 1047-3211 10.1093/cercor/bhs352 23146964 PMC3920766

[70] Naci L. et al., “A common neural code for similar conscious experiences in different individuals,” Proc. Natl. Acad. Sci. U. S. A. 111(39), 14277–14282 (2014). 10.1073/pnas.1407007111 25225384 PMC4191782

[71] Naci L. Sinai L. Owen A. M., “Detecting and interpreting conscious experiences in behaviorally non-responsive patients,” Neuroimage 145, 304–313 (2017). NEIMEF 1053-8119 10.1016/j.neuroimage.2015.11.059 26679327

[72] Kirilina E. et al., “The physiological origin of task-evoked systemic artefacts in functional near infrared spectroscopy,” Neuroimage 61(1), 70–81 (2012). NEIMEF 1053-8119 10.1016/j.neuroimage.2012.02.074 22426347 PMC3348501

[r73] Tachtsidis I. Scholkmann F., “False positives and false negatives in functional near-infrared spectroscopy: issues, challenges, and the way forward,” Neurophotonics 3(3), 031405 (2016). 10.1117/1.NPh.3.3.031405 27054143 PMC4791590

[74] Huppert T. J. et al., “Sensitivity of neural-hemodynamic coupling to alterations in cerebral blood flow during hypercapnia,” J. Biomed. Opt. 14(4), 044038 (2009). JBOPFO 1083-3668 10.1117/1.3210779 19725749 PMC2774124

