# Supplementary material for: Revealing the spatiotemporal requirements for accurate subject identification with resting-state functional connectivity: a simultaneous fNIRS-fMRI study
Source: Neurophotonics. 2023 Feb 3;10(1):013510. doi: 10.1117/1.NPh.10.1.013510 (PMC9896013; doi:10.1117/1.NPh.10.1.013510)
Supplement: Supplementary file 1 [file NPh_010_013510_SD001.docx]

**Revealing the spatiotemporal requirements for accurate subject identification with resting-state functional connectivity: a simultaneous fNIRS-fMRI study**

Sergio L. Novi^1,2^, Alex C. Carvalho^1,3^, R. M. Forti^1,4^, Fernado Cendes^5,6^, Clarissa L. Yasuda^3,5,6^, Rickson C. Mesquita^1,5^

^1^ ^“^Gleb Wataghin” Institute of Physics, University of Campinas, Campinas, SP 13083-859 (Brazil)

^2^ Department of Physiology and Pharmacology, Western University, London, ON N6A 5C1 (Canada)

^3^ Laboratory of Neuroimaging, University of Campinas, Campinas, SP 13083-888 (Brazil)

^4^ Division of Neurology, The Children’s Hospital of Philadelphia, PA, 19104, Philadelphia, USA

^5^ Brazilian Institute of Neuroscience and Neurotechnology (BRAINN), Campinas 13083-888, Brazil

^6^ Department of Neurology, School of Medical Sciences, University of Campinas (UNICAMP), Campinas 13083-888, Brazil

Corresponding author:

Sergio L. Novi: [sjunior@uwo.ca](mailto:sjunior@uwo.ca)

**SUPPLEMENTARY MATERIAL**


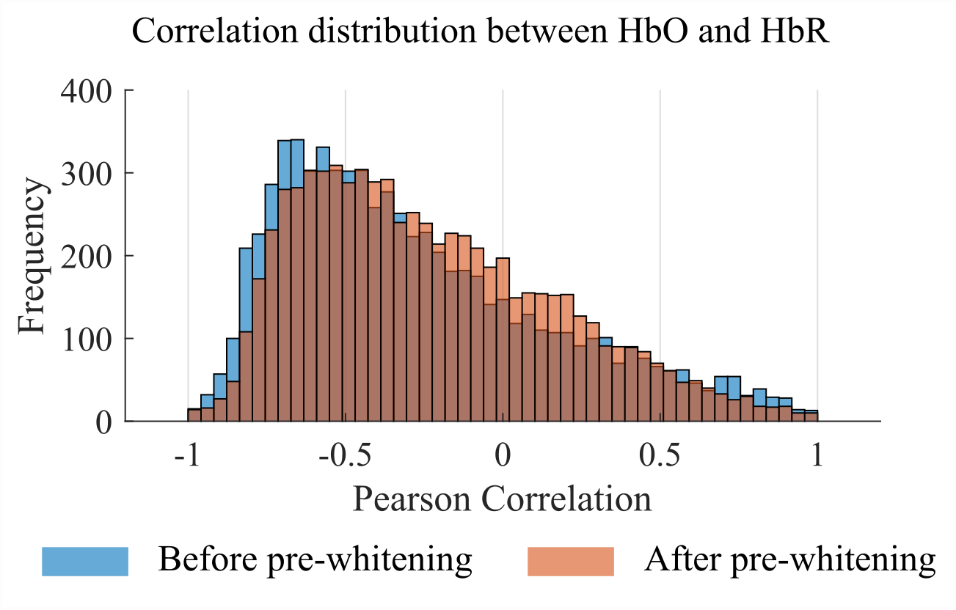


**Figure S1:** Pearson correlation coefficient between HbO and HbR for the same fNIRS channel for all channels. The pre-whitening step did remove the expected anti-correlation between HbO and HbR.


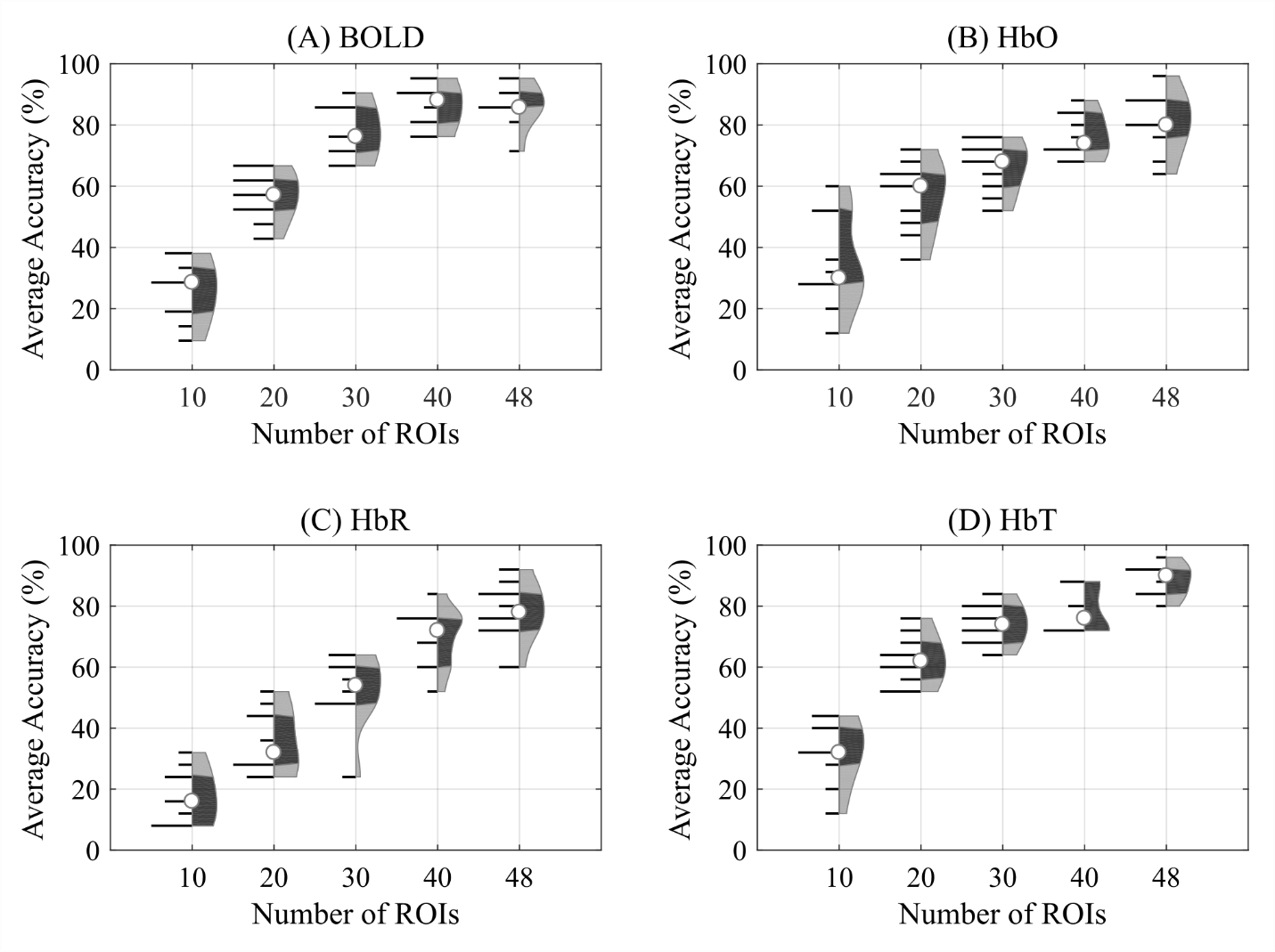


**Figure S2:** Average geodesic classification accuracy as a function of the number of regions of interest (ROIs) when only one run was used for training and another for testing for the BOLD (A), HbO (B), HbR (C), and HbT(D) contrasts. Each distribution has 10 values of average correlation from 300 repetitions each. The performance on subject identification increases with the number of ROIs used in the classification for all contrasts (BOLD, HbO, HbR, and HbT); in particular, HbT accuracy performed similarly to BOLD when the number of ROIs across the two techniques was equal.


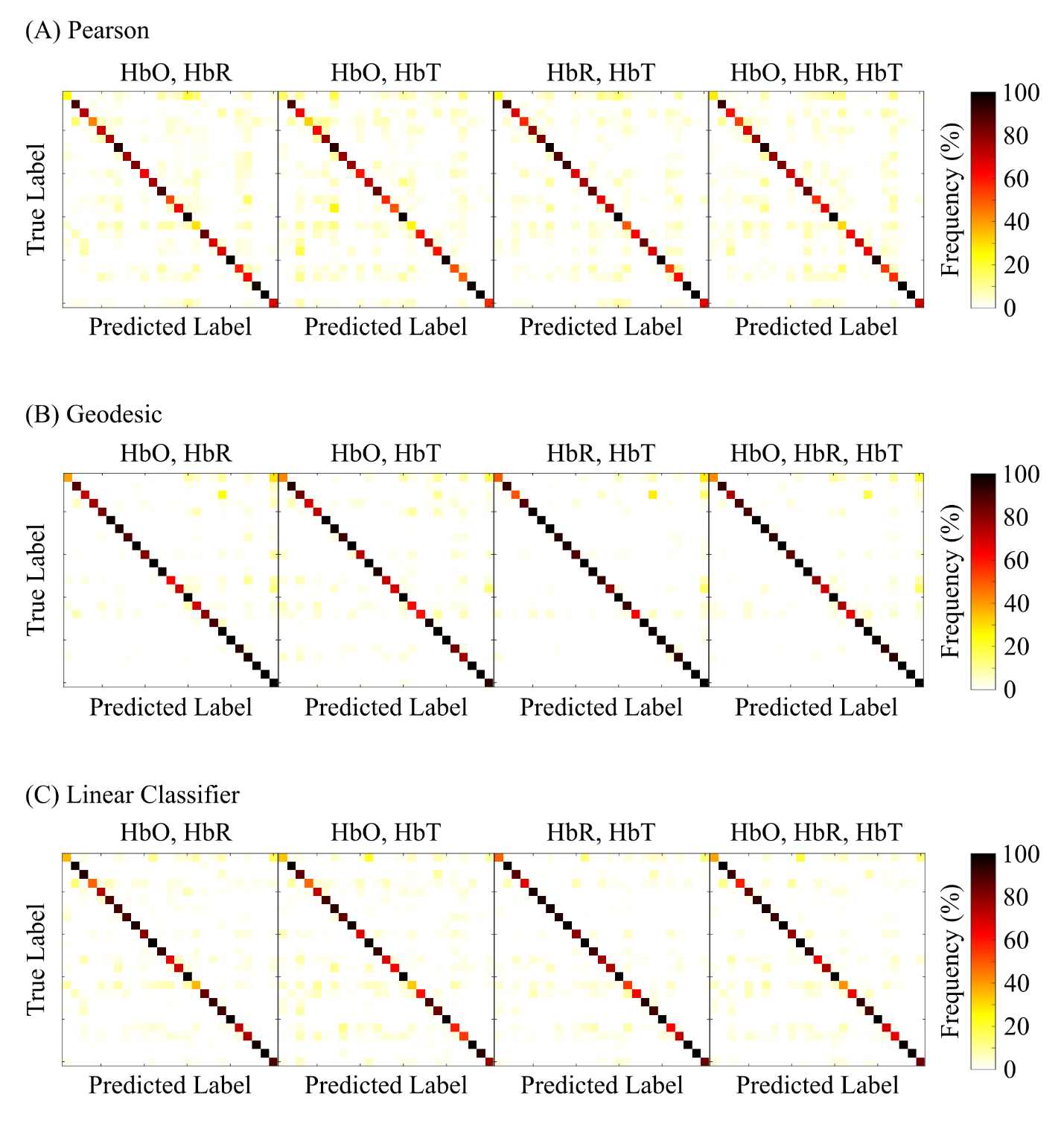


**Figure S3:** Confusion matrices during subject identification when different fNIRS contrasts are combined. In each subplot, the vertical label indicates the correct label of each participant. The horizontal label shows the label that was predicted from each classifier. A perfect classifier would have only non-zero elements equal to 100% in the main diagonal. The maximum possible frequency in number of occurrences is 300.
